# Supplementary material for: Synergism of Photo-Induced Electron Transfer and Aggregation-Induced Quenching Mechanisms for Highly Sensitive Detection of Silver Ion and Captopril
Source: Molecules. 2023 Apr 22;28(9):3650. doi: 10.3390/molecules28093650 (PMC10180011; doi:10.3390/molecules28093650)
Supplement: Supplementary file 1 [file molecules-28-03650-s001.zip › molecules-2278920-supplementary.pdf]

Supporting Information

For

Synergism of Photo-Induced Electron Transfer and Aggregation-Induced Quenching Mechanisms for Highly Sensitive Detection of Silver Ion and Captopril

Jing Zhu [1], Lei Hu [1], Xiangying Meng [2,*], Feng Li [1], Wenjuan Wang [1], Guiyang Shi [1] and Zhongxia Wang [1,*]

1 School of Chemistry and Chemical Engineering, Yancheng Institute of Technology, Yancheng 224051, China
2 School of Medical Laboratory, Weifang Medical University, Weifang 261053, China
* Correspondence: mengxy2015@wfmc.edu.cn (X.M.); angzx198411@nju.edu.cn (Z.W.)

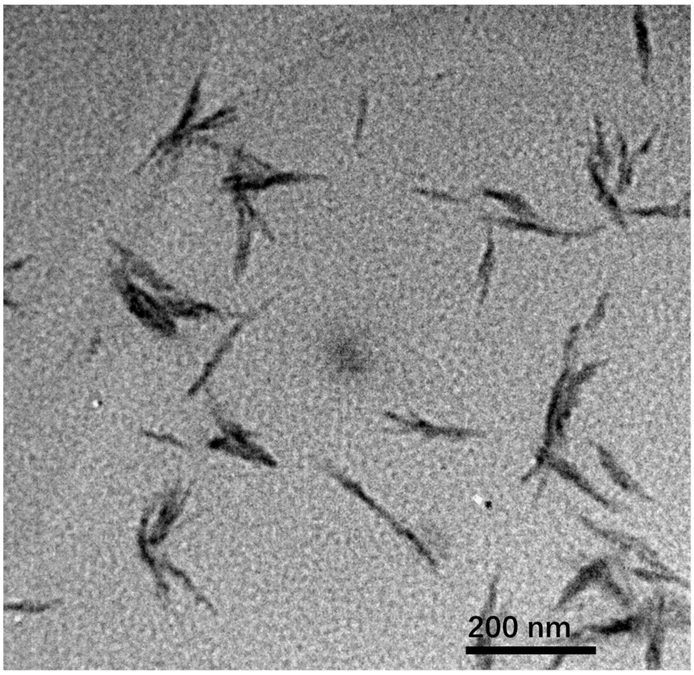

Figure S1 Enlarged-TEM image of ONCNs.

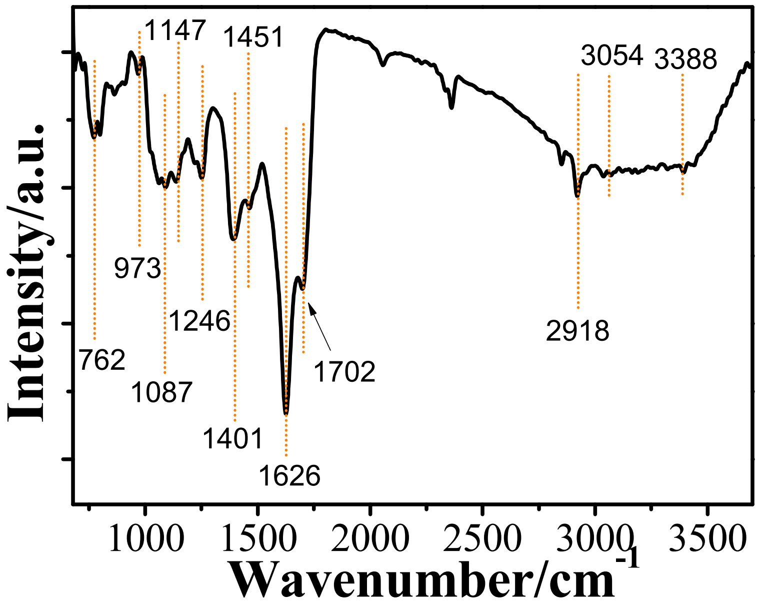

Figure S2 FT-IR spectrum of ONCNs.

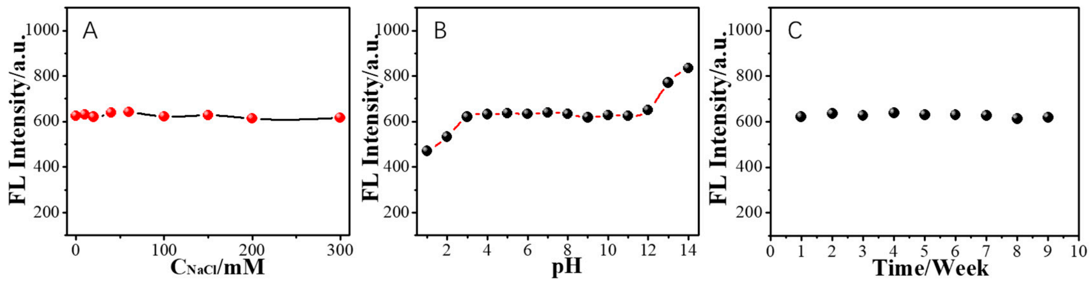

Figure S3 FL intensity at 390 nm (excitation at 335 nm) of the ONCNs as a function of A) NaCl concentration, B) solution pH value, and C) storage time. the excitation and emission slit widths were 5 nm and 3 nm, respectively.

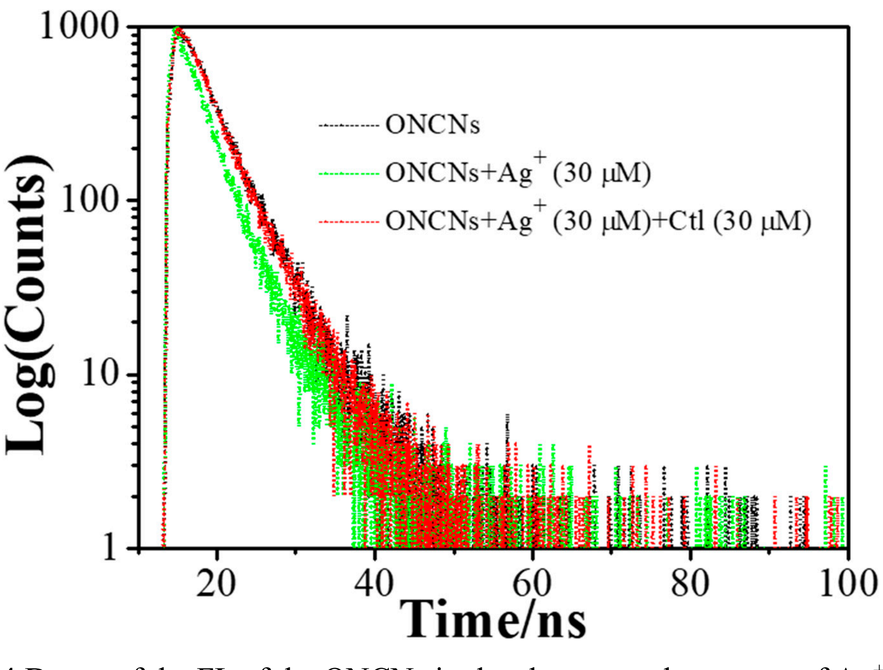

Figure S4 Decay of the FL of the ONCNs in the absence and presence of Ag^+ (30 μM) or Ag^+ (30 μM) and Ctl (30 μM).

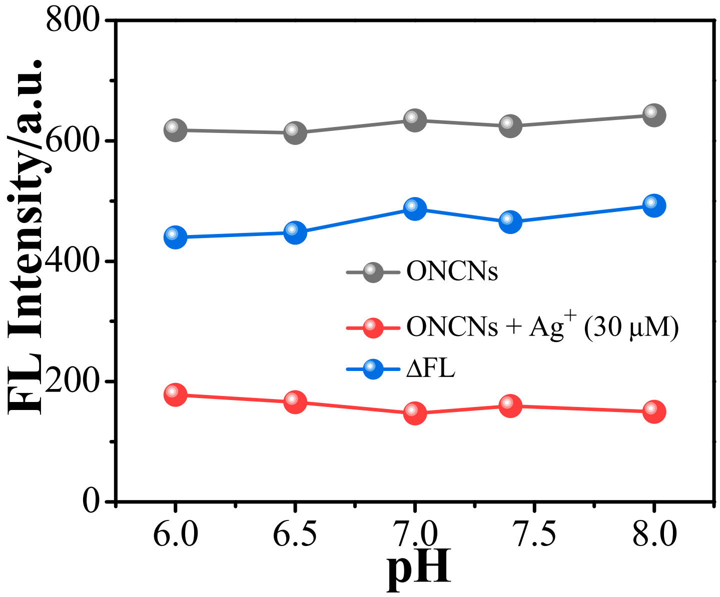

Figure S5 The FL intensity of the ONCNs (10 μg mL^{-1}) in different pH values of Tris-HNO_3 buffer solution in the absence or presence of Ag^+ ions (30 μM).

As shown Figure S5 (blue line), it can clearly be seen that the relative FL of ONCNs efficiency increased gradually as the pH increased from 6.0 to 7.0, then, the relative FL value decreased gradually as the pH increased from 7.0 to 8.0. This would mean that there would be strong interactions between the Ag^+ ion and the ONCNs at acid-base solution, meaning that Ag^+ ion would decrease the FL of the ONCNs efficiently. On the other hand, Ag^+ ion can complex with OH^- to form the insoluble hydrated oxide Ag(OH) under strong alkaline conditions, preventing coordination of Ag^+ ion to the ONCNs, leading to incomplete FL quenching due to the suppression of ONCNs-Ag^+ aggregate formation. Considering the protonation-deprotonation of the ONCNs and the stable effect of the Ag^+ ion for pH values, pH 7.0 Tris-HNO_3 buffer was selected as the optimum solvent for sensor performance.

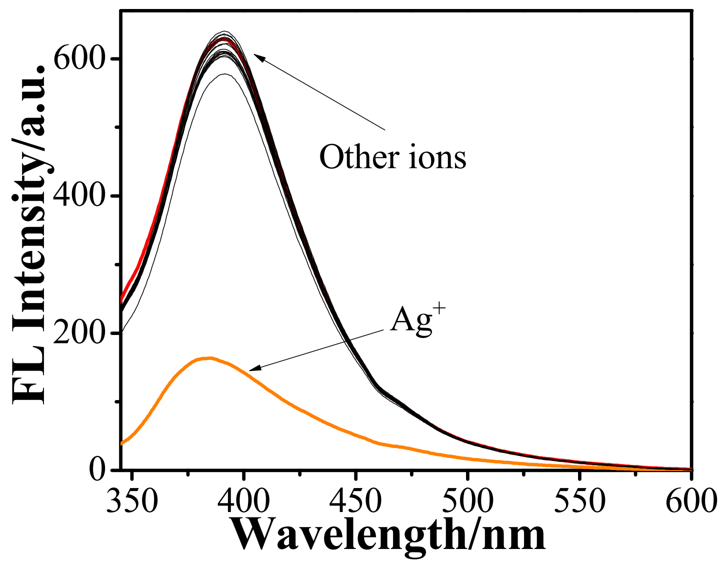

Figure S6 FL spectra of ONCNs in the presence of various metal ions. The concentration of Ag^+ ions were 30 μM, that of Hg^{2+} was 50 μM, and those of the other metal ions were 500 μM. Excitation/emission wavelengths at 335/390 nm, and both the excitation and emission slit widths were 5 nm and 3 nm, respectively.

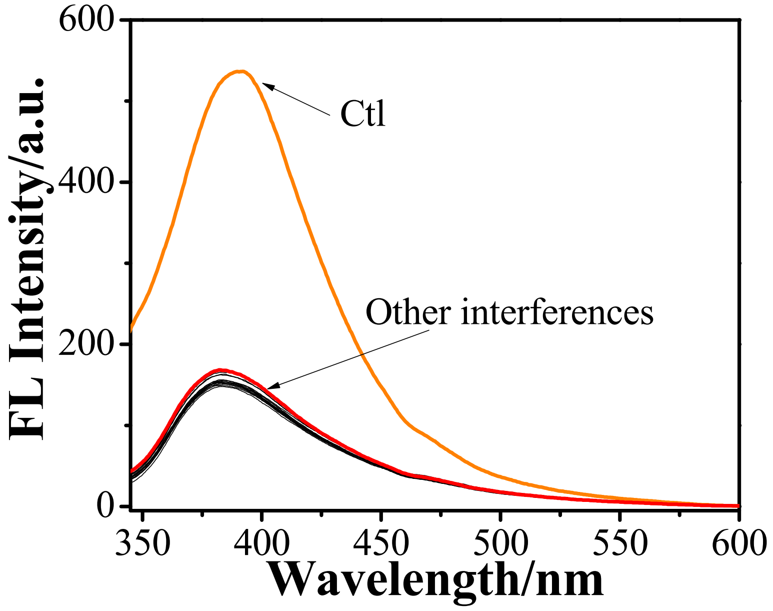

Figure S7 FL spectra of ONCNs-Ag^+-based in the presence of various biomolecules. The concentration of Ctl was 30 μM, that of dopamine, adrenaline and serotonin were 50 μM, respectively, and those of the other biomolecules were 500 μM. Excitation/emission wavelengths at 335/390 nm, and both the excitation and emission slit widths were 5 nm and 3 nm, respectively.

Table S1. Comparison of different methods and/or probes for Ag^+ ion determination.

Analyte	Methods	Probe	Linear range	Detect limit	Refs.
**Ag^+ ion**	Fluorescence	FITC-CdTe QDs	0.125-12.5 μM	40 nM	[1]
**Ag^+ ion**	Fluorescence	BSA-Au NCs	5.0 - 60.0 μM	0.23 μM	[2]
**Ag^+ ion**	Fluorescence	CDs	5 - 100 μM	1.40 μM	[3]
**Ag^+ ion**	Fluorescence	ONCNs	3 - 30 μM	0.78 μM	Our method

Table S2. Comparison of different methods and/or probes for Ctl determination.

Analyte	Methods	Probe	Linear range	Detect limit	Refs.
**Ctl**	Fluorescence	PCNBPs	0.05 - 40 μM	15.9 nM	[4]
**Ctl**	Fluorescence	MoOx QDs	1 - 40 μM	0.51 μM	[5]
**Ctl**	Fluorescence	B,N-CQDs	0.1 - 60 μM	30 nM	[6]
**Ctl**	Fluorescence	ONCNs	1 - 30 μM	74 nM	Our method

References

1. Ting Liu, Li-Xing Fu, Chen-Hui Yin, Meng Wu, Li-Gang Chen, Na Niu, Design of smartphone platform by ratiometric fluorescent for visual detection of silver ions, Microchemical Journal, 2022, 174, 107016.

2. Zheng-Fen Pu, Jun Peng, Qiu-Lin Wen, Yu Li, Jian Ling, Peng Liu, Qiu-E Cao, Photocatalytic synthesis of BSA-Au nanoclusters with tunable fluorescence for highly selective detection of silver ion, Dyes and Pigments, 2021, 193, 109533.

3. Yun-Xia Ma, Wen-Juan Lv, Yong-Lei Chen, Min Na, Juan-Juan Liu, Yang-Xia Han, Su-Dai Ma, Xing-Guo Chen, Facile preparation of orange-emissive carbon dots for the highly selective detection of silver ions, 2019, New Journal of Chemistry, 43, 5070-5076.

4. Zhong-Xia Wang, Yuan-Fei Gao, Xian-He Yu, Fen-Ying Kong, Wei-Xin Lv, Wei Wang, Photoluminescent coral-like carbon-branched polymers as nanoprobe for fluorometric determination of captopril, Microchimica Acta, 2018, 9, 185.

5. Sai-Jin Xiao, Xiao-Jing Zhao, Zhao-Jun Chu, Han-Xu, Guo-Qing Liu, Cheng-Zhi Huang, Li Zhang, New off-on sensor for captopril sensing based on photoluminescent MoOx quantum dots, ACS Omega 2017, 2, 1666-1671.
6. Xiao-Hua Jiang, Dong-Miao Qin, Gui-Chun Mo, Jin-Su Feng, Xiang-Fei Zheng, Bi-Yang Deng, Facile preparation of boron and nitrogen co-doped green emission carbon quantum dots for detection of permanganate and captopril, Analytical Chemistry, 2019, 91, 11455-11460.
